# Gut Microbiota and Respiratory Infections: Insights from Mendelian Randomization

**DOI:** 10.3390/microorganisms11082108

**Published:** 2023-08-18

**Authors:** Shengyu Huang, Jiaqi Li, Zhihao Zhu, Xiaobin Liu, Tuo Shen, Yusong Wang, Qimin Ma, Xin Wang, Guangping Yang, Guanghua Guo, Feng Zhu

**Affiliations:** 1 Medical Center of Burn Plastic and Wound Repair, The First Affiliated Hospital of Nanchang University, Nanchang 330006, China; 15070440503@163.com (S.H.); sylvialjq@163.com (J.L.); ncdxzzh@163.com (Z.Z.); 18772407841@163.com (X.W.); 18810643951@163.com (G.Y.); 2 Department of Critical Care Medicine, Shanghai East Hospital, Tongji University School of Medicine, Shanghai 200120, China; drlxb@outlook.com (X.L.); st516033612@163.com (T.S.); maqimin0311@163.com (Q.M.); 3 ICU of Burn and Trauma, Changhai Hospital, Shanghai 200433, China; yusquirrelw@163.com

**Keywords:** gut microbiota, respiratory tract infections, microbial metabolites, gut–lung axis

## Abstract

The role of the gut microbiota in modulating the risk of respiratory infections has garnered increasing attention. However, conventional clinical trials have faced challenges in establishing the precise relationship between the two. In this study, we conducted a Mendelian randomization analysis with single nucleotide polymorphisms employed as instrumental variables to assess the causal links between the gut microbiota and respiratory infections. Two categories of bacteria, family *Lactobacillaceae* and genus *Family XIII AD3011*, were causally associated with the occurrence of upper respiratory tract infections (URTIs). Four categories of gut microbiota existed that were causally associated with lower respiratory tract infections (LRTIs), with order *Bacillales* and genus *Paraprevotella* showing a positive association and genus *Alistipes* and genus *Ruminococcaceae UCG009* showing a negative association. The metabolites and metabolic pathways only played a role in the development of LRTIs, with the metabolite deoxycholine acting negatively and menaquinol 8 biosynthesis acting positively. The identification of specific bacterial populations, metabolites, and pathways may provide new clues for mechanism research concerning therapeutic interventions for respiratory infections. Future research should focus on elucidating the potential mechanisms regulating the gut microbiota and developing effective strategies to reduce the incidence of respiratory infections. These findings have the potential to significantly improve global respiratory health.

## 1. Introduction

The human gastrointestinal tract serves as a habitat for trillions of microorganisms, including bacteria, fungi, viruses, and parasites [1]. This dynamic and balanced ecosystem plays a vital role in various biological activities within the human body, such as digestion, metabolism, and inflammation, by regulating multiple endocrine, neural, and immune pathways [2,3]. Clinically, various factors can contribute to the dysregulation of gut microecology, such as aggressive pathogens, prolonged use of antibiotics, physical mucosal damage, extended fasting, and genetic factors [4,5]. The dysbiosis of the gut microbiota, particularly the decrease in obligate anaerobes in critically ill patients, increases the risk of infection [6], with respiratory infections being recognized as the most prevalent infectious disease worldwide and as a leading cause of incidence and mortality. Previous studies have revealed that some microbiota influence susceptibility to pathogens through the gut–lung axis [7]. Moreover, several clinical studies have demonstrated the effectiveness of oral probiotics, such as *Lactobacillus plantarum* and *Lactobacillus casei rhamnosus*, in reducing the incidence of ventilator-associated pneumonia, acute upper respiratory tract infections, and COVID-19 infections [8,9,10]. Consequently, it becomes plausible to consider monitoring respiratory tract infections by targeting the flora [11,12]. However, due to the challenges associated with conducting high-quality clinical trials, confirming this causal relationship in clinical patients has been difficult. Moreover, the identification of other specific intestinal flora that may be potentially causally involved in the development of respiratory infections poses challenges when relying solely on traditional methods.

Mendelian randomization (MR) is an emerging statistical method in epidemiology that uses genetic variation as an instrumental variable to explore the causal relationship between exposure and outcome [13]. Single nucleotide polymorphisms (SNPs) refer to the DNA sequence diversity caused by a variation in a single nucleotide at the genomic level and remain unaffected by acquired confounders [14]. Utilizing SNPs as instrumental variables provides several advantages over randomly assigning exposures in artificial designs or traditional randomized controlled trials (RCTs) [15]. This approach allows us to assess the effect of the microbiota on infection while minimizing the impact of confounding factors.

In this study, we employed large-scale genome-wide association studies (GWAS) to analyze the influence of gut microbiomics and metabolomics on the incidence of respiratory tract infections. Following the guidelines outlined in the “STROBE-MR” (Strengthening the Reporting of Observational Studies in Epidemiology-Mendelian Randomization) guidelines [16], we performed a microbiomics analysis using two-sample MR methods. Our objective was to identify specific microbial species that may serve as modifiers in the context of susceptibility to infection.

## 2. Materials and Methods

### 2.1. Source of Data on Exposure and Outcome

**Figure 1:**
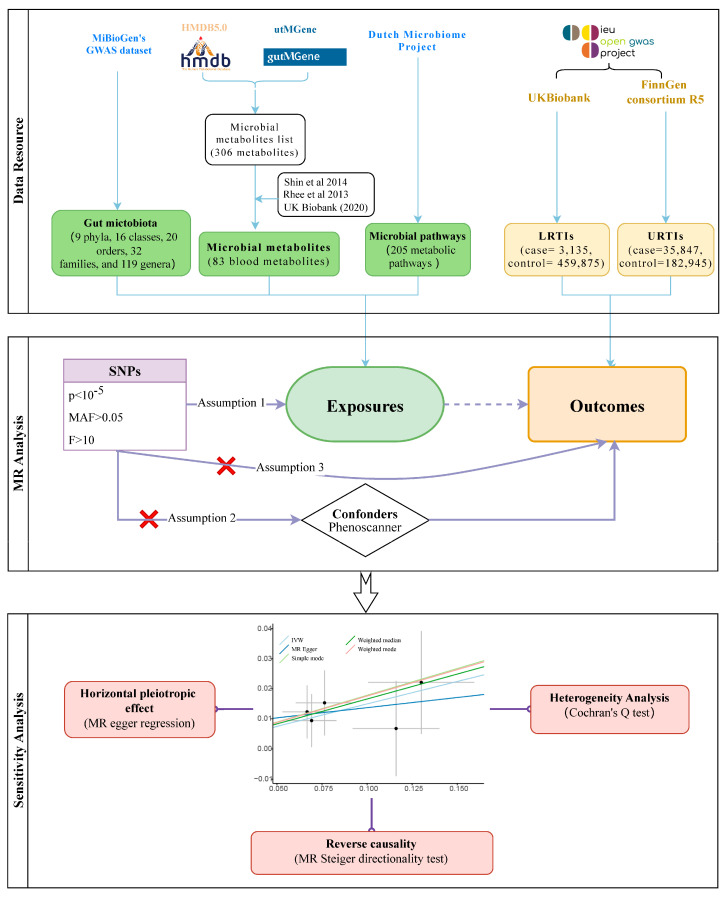
Flow chart of this study.

In our study, the exposure variable consisted of the gut microbiota, metabolites, and functional pathways, while the outcome variable was respiratory tract infections (Figure 1). The abundance data concerning the gut microbiota were obtained from MiBioGen’s GWAS dataset [17], which comprised a meta-analysis of 24 cohorts and a total of 18,340 individuals. This dataset included fecal microbiota data for various taxonomic categories (9 phyla, 16 classes, 20 orders, 32 families, and 119 genera) based on the 16S rRNA gene amplicon sequences. After removing unknown species, a total of 122,110 associated SNPs were retained for analysis. For the gut microbial metabolites, we searched the online databases, namely the Human Metabolome Database (HMDB5.0) [18] and utMGene [19], resulting in the identification of 316 blood microbial metabolites. Additionally, we matched the latest GWAS data from three pooled cohorts: a study comprising more than 7824 adults that investigated over 400 metabolites in blood [20], metabolic biomarker data from Nightingale Health in the UK Biobank (2020), and data from the Framingham Heart Study involving 2076 participants [21]. Through this data-matching process, we obtained 83 relevant metabolites for further analysis. Summary data concerning the microbial pathways were primarily derived from the Dutch Microbiome Project, which investigated the composition and function of the gut microbiome in 8208 individuals [22]. This analysis included 205 possible metabolic pathways related to microbial function.

GWAS summary data concerning the outcome factors were obtained from the online database MRC IEU OpenGWAS [23]. The GWAS of LRTIs (case = 3135, control = 459,875) were obtained from the UK Biobank dataset and of URTIs (case = 35,847, control = 182,945) were from the FinnGen consortium R5. Both the exposed and outcome populations were from the Europe.

### 2.2. MR Analysis

Instrumental variables (IVs) must satisfy three assumptions [16]. (1) Relevance assumption: IVs are related to the exposure studied. (2) Independence assumption: IVs are independent of possible confounding factors. (3) Exclusion assumption: IVs do not directly affect the outcome; they can only affect the outcome by influencing exposure factors.

We followed three core assumptions to identify the IVs we needed. First, setting a statistical significance threshold of 1 × 10^−5^ [24], screening out genetic variant SNPs that were strongly associated with the microbe but not directly associated with URTIs and LITIs, and excluding minor allele frequencies (MAFs) with a threshold of 0.01. We assessed the power of the instrumental variables using the F value [25], *F = R^2^ × (N – 1 − i)/(1 − R^2^) × i*, and IVs with an F less than 10 were excluded. Where *N* is the sample size, *i* is the number of valid SNPs, *R^2^ = 2 × EAF × (1 − EAF) × β^2^(i < 10)* or *R^2^ = 2 × EAF × (1 − EAF) × β^2^/((2 × EAF × (1 − EAF) × β^2^) + (2 × EAF × (1 − EAF) × N × SE^2^))(i ≥ 10)* [26]. In addition, considering that linkage disequilibrium may exist between IVs, we excluded them based on a linkage disequilibrium parameter of 0.001 and a genetic distance of 10,000 kb [27]. Secondly, the online site PhenoScanner was used to screen for possible confounders with the IVs (HDL cholesterol, cigarette, body mass index, and body fat percentage) and to prevent these factors from interfering with the effect of exposure on the results [28].

To assess the genetically predicted specific association between the gut microbiota abundance and respiratory tract infections, we employed five methods (inverse variance weighted, MR Egger, simple mode, weighted median, and weighted mode). The inverse variance weighted (IVW) method served as the main fixed-effects meta-analysis, while the remaining four methods were utilized for secondary validation, reinforcing the reliability of the results. The IVW method was used as the primary MR method, and results with an IVW < 0.05 were taken as initial positives.

### 2.3. Sensitivity Analysis and Reverse Causation

To further explore the heterogeneity of the results, a sensitivity analysis was performed to assess the heterogeneity among the SNPs associated with each microbial unit via Cochran’s Q test [29,30], and heterogeneity was indicated if the Q value was less than 0.05. Using MR Egger regression, we could assess whether genetic instruments have a horizontal pleiotropic effect on outcomes [31]. Finally, to ensure there is reverse causality, we used the MR Steiger directionality test [32]. It calculates the variance explained in the exposure and the outcome by the instrumenting SNPs, and it tests if the variance in the outcome is less than in the exposure. When testing multiple exposure factors together, there is an increased risk of a type I statistical error (α). To minimize this, we applied the Bonferroni correction to adjust the test levels. The Bonferroni correction divides the desired significance level (P) by the number of comparisons made (n) to maintain an appropriate overall significance level [33]. In our study, we conducted the Bonferroni correction for the analysis of the gut microbial abundance based on their classification (order: 0.05/1; family: 0.05/3; genus: 0.05/15). Furthermore, for the analysis of the microbial metabolites, we applied the Bonferroni correction by setting the corrected *p*-value to 0.05 divided by 9, while the microbial pathway was 0.05/20.

All the data analyses were based on the R package (4.2.3): Two Sample MR0.5.7 [32] and PhenoScanner [34].

## 3. Results

### 3.1. Gut Microbiota Abundance and Infection

#### 3.1.1. URTIs

**Figure 2:**
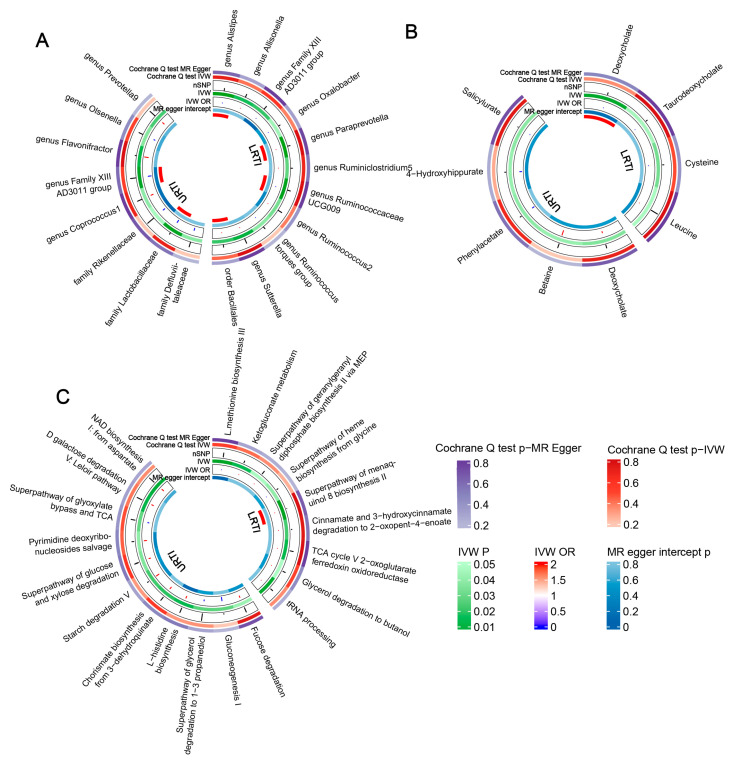
Circular heat map of LRTIs and URTIs with an MR IVW *p* less than 0.05. (**A**) is the gut microbiota abundance; (**B**) is the microbial metabolites; and (**C**) is the microbial pathways.

After applying the IVW method to assess the association between the gut microbiota abundances and URTIs, a total of eight flora abundances were identified as being initially significantly associated (*p* < 0.05). Subsequently, the causality of the family *Rikenellaceae* was further confirmed via the MR Egger and weighted median methods. Additionally, the weighted median method supported the causality of the family *Lactobacillaceae* and genus *Flavonifractor* (Figure 2A, Supplementary Table S1).

**Figure 3:**
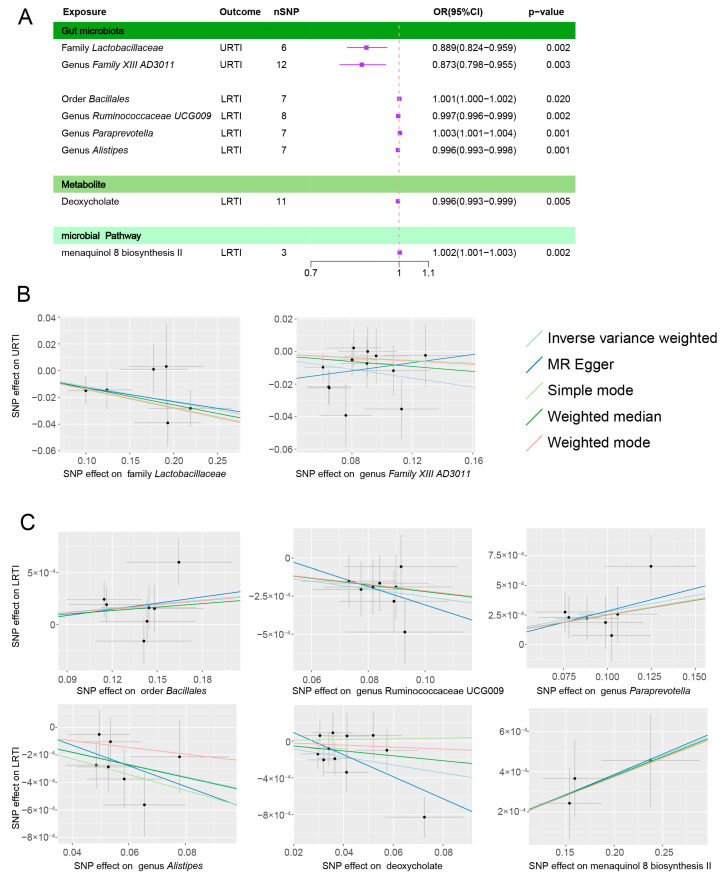
Positive results after the Bonferroni correction. (**A**) is the forest plots with significant results after the Bonferroni correction; (**B**) is the scatter plots of the relationship between the positive exposures and URTIs after correction; and (**C**) is the scatter plots of the relationship between the positive exposures and LRTIs after correction.

However, after conducting the Bonferroni multiple corrections, only the family *Lactobacillaceae* (OR = 0.889, 95% CI: 0.824–0.959) and genus *Family XIII AD3011* (OR = 0.873, 95% CI: 0.798–0.955) remained significantly and causally related to URTIs (Table 1). Both showed a negative correlation with the occurrence of URTIs (Figure 3A,B).

#### 3.1.2. LRTIs

An initial causal relationship between 11 microbial genera and LRTIs was found, including 1 order and 10 genera, and the weighted median further verified that there was a statistically significant causal relationship between the genus *Alistipes*, genus *Paraprevotella*, genus *Ruminococcaceae UCG009* and genus *Ruminococcus torques* groups (Figure 2A, Supplementary Table S1).

The results after multiple corrections still showed a causal relationship between the four related groups (Table 1), with the order *Bacillales* (OR = 1.001, 95% CI: 1.0002–1.002) and genus *Paraprevotella* (OR = 1.003, 95% CI: 1.001–1.004) showing a positive correlation, while the genus *Alistipes* (OR = 0.996, 95% CI: 0.993–0.998) and genus *Ruminococcaceae UCG009* (OR= 0.997, 95%CI: 0.996–0.999) were negatively correlated (Figure 3A,C).

### 3.2. Microbial Metabolites, Pathways, and Infections

A total of 83 accessible metabolites and 205 pathways to identify the key targets were included. If only considering the *p* value of the IVW, there were 9 metabolites (Supplementary Table S2) and 20 relevant functional pathways (Supplementary Table S3) that seemed initial works (Figure 2B,C). However, only deoxycholate (OR = 0.996, 95% CI: 0.993–0.999) and the superpathway of menaquinol 8 biosynthesis II (OR = 1.002, 95% CI: 1.001–1.003) were positively correlated with the incidence of ALRI after multiple corrections (Table 1, Figure 3A,C).

### 3.3. Heterogeneity Test and Reverse Causality

A sensitivity analysis was performed for all the results. When using the MR Egger regression intercept method, no evidence of the multiplicity of the exposure factor levels was found, *p* > 0.05. Using Cochran’s Q test, no heterogeneity was found either, with *p* > 0.05. To further verify whether there was an inverse causal relationship between these florae and infections, we applied the MR Steiger directionality test. There was no reverse causality between the exposures and various infections (Table 1, Supplementary Tables S1–S3).

## 4. Discussion

We have made significant strides in identifying specific gut microbiota that exert a causal effect on the infection risk. The abundance of these microbiota has been shown to play a pivotal role in either enhancing or diminishing the likelihood of infection. Notably, the gastrointestinal and respiratory tracts share similarities in terms of the anatomical structure and functional characteristics. This parallelism in early microbial colonization of both sites facilitates a close interplay, particularly concerning colonization resistance mechanisms [11].

The gut–lung axis serves as a vital conduit through which the gut microbiota influences susceptibility to respiratory infections. This axis operates remotely, with the immune response and microbial-associated molecular patterns playing integral roles [35,36]. However, despite the progress made, the precise underlying mechanisms require further investigation. Although existing studies have provided valuable insights, multiple potential pathways warrant consideration.

The foremost gut microbiota is involved in maintaining the gastrointestinal mucosal barrier and restricting the proliferation and spread of pathogens. Symbiotic bacteria exert their protective effects by outcompeting pathogenic bacteria for nutrients, altering the intestinal microenvironment, inducing the production of antimicrobial factors in the intestinal epithelium, and releasing metabolites such as short-chain fatty acids, polyamines, and bile acids that enhance the barrier function of the intestinal mucosa [37,38]. This phenomenon, known as colonization resistance, helps prevent the colonization of pathogenic bacteria [39]. For instance, *Bifidobacteria* reduce the intestinal pH through lactose fermentation, thus inhibiting the colonization of pathogenic *E. coli* [40]. Disruption of the microbial composition can compromise the integrity of the mucosal barrier and increase intestinal permeability, allowing bacteria to translocate and reach the mesenteric lymph nodes or even distant organs [41,42]. Such remote crosstalk may disrupt the original colonization resistance in the respiratory tract, leading to infection [43,44].

In addition to regulating the maintenance of intestinal epithelial cell turnover and barrier function, microbial metabolites in healthy patients are involved in the regulation of immune responses and inflammation [45]. Dysbiosis of the gut microbiota may downregulate the immune recognition mechanisms in the lungs, thereby reducing the ability to clear viruses from the lungs [46]. From the perspective of local immunity in the respiratory mucosa, gut microbes can influence the function of epithelial cells, macrophages, and dendritic cells in the respiratory tract, driving IFN signaling to restrict pathogen replication [36,46,47]. In mouse experiments, dysbiosis of the gut microbiota altered the function of the pulmonary mucosa-associated invariant T MAIT cells, leading to increased early colonization of the lungs by *Mycobacterium tuberculosis* [44]. IL-17A and IL-22 may also be important mediators of the association, with the former triggering an increase in pulmonary GM-CSF stimulating alveolar macrophages to kill and clear pathogens [48], and symbiotic bacteria stimulating the transfer of IL-22-producing group 3 innate lymphocytes to the lungs to exert anti-pneumonia effects [49]. Furthermore, the transmission through the blood and lymphatic system is linked to a systemic cellular response. For example, short-chain fatty acids regulate the formation of pro-inflammatory cytokines such as TNF-α, IL-12, and IL-10 by activating innate immune neutrophils, macrophages, and dendritic cells [42,50]. They also contribute to the formation of an anti-inflammatory environment by inhibiting NF-κB in B cells and promoting the production of extra-thymic regulatory T cells, thus limiting the inflammatory process [51,52].

It is important to acknowledge that the human body operates as a complex system, and the relationship between the gut microbiota and infection is not a simple one-way interaction. Existing infections can disrupt gut microbiota homeostasis, thereby exacerbating pulmonary infections and potentially leading to sepsis, forming a detrimental cycle [53]. Previous treatments of respiratory tract infections have often emphasized the modulation of gut microbiota abundance [54]. However, the efficacy of common probiotic interventions has shown inconsistent results [55,56]. Therefore, identifying specific targets to modulate the gut microbiota may hold promise for disease treatment. Our study stands as the first to utilize genetic variation as an instrumental variable in assessing the potential causal relationship between individual gut microbiota and respiratory infections. This approach offers novel insights into the complex interplay between the gut microbiota and respiratory health.

In this study, we conducted separate analyses for URTIs and LRTIs, taking into consideration the differences in the microbial communities originally colonized in these regions [57]. In healthy individuals, the microbial abundance in the LRT is typically lower compared to the URT [58]. The URT, being more exposed to the external environment, is colonized by a diverse range of microbial species soon after birth, with *Actinobacteria* being a dominant phylum [59,60]. On the other hand, the microbial composition of the LRT is more variable due to the unique physiological environment characterized by factors such as the oxygen partial pressure, pH, and temperature. These environmental differences may influence the selection and growth of microbiota, leading to variations in the microbial composition [61]. In the LRT, the phyla *Bacteroidetes* and *Firmicutes* are typically abundant, and there is a notable genus abundance, including *Prevotella* and *Veillonella* [62]. Likewise, in our results, the microbes affecting their susceptibility were inconsistent. For the incidence of URTIs, only the family *Lactobacillaceae* and genus *Family XIII AD3011* had a negative relationship. *Lactobacillaceae* are consistently considered to be beneficial bacteria in the human body [63]. In children with recurrent respiratory infections, a microbiota imbalance is manifested by a significant decrease in the number of *bifidobacteria* and *lactobacilli* [54]. Oral intestinal probiotics have been proven to prevent bacterial pneumonia and help accelerate recovery from respiratory viral infections. A meta-analysis of 12 clinical trials showed that probiotics containing *Lactobacillus* prevent the incidence of URTIs [9]. In the prospective study, long-term consumption of dairy products containing *Lactobacillus casei* could also reduce the incidence of URTIs. These beneficial effects may be attributed to the ability of *Lactobacillus* to stimulate the release of cytokines such as IL-4 and IL-10 and the effective enrichment of IL-12, INF-γ, and TNF α in mediastinal lymph nodes, which help limit the systemic spread of bacteria [64,65]. Moreover, in vitro, studies have also shown that *Lactobacillus* and *Bifidobacterium* have inhibitory effects on pathogenic bacteria, including *Pseudomonas aeruginosa*, *Escherichia coli*, and *Klebsiella pneumoniae* [66]. Among the microbiota associated with LRTIs, order *Bacillales* and genus *Paraprevotella* increase the risk, while genus *Alistipes* and genus *Ruminococcaceae UCG009* may have a protective effect. Although the mechanisms involved are still unknown, there is some clinical evidence to support them. In studies of respiratory flora in patients with ventilator-associated pneumonia, *Bacillales* were positively associated with multiple microbial alterations [67]. Moreover, in mouse models, *Bacillales* abundance also showed an increase in mice with high-calorie diets and LPS-induced pneumonia [68]. *Prevotella*, a common symbiotic bacterium in the lower respiratory tract, has been implicated in the pathogenesis of several inflammatory diseases due to stimulating local and systemic immune responses [69,70]. Additionally, *Prevotella* has been found to be enriched in the intestine following infections with SARS-CoV-2 or tuberculosis, while *Alistipes* abundance has been observed to be reduced in these infections [71].

Metabolites are thought to play an important role in the gut–organ axis, and we selected 76 blood microbial metabolites that contained GWAS data. We only found a directional relationship between the blood levels of deoxycholate and LRTIs. Deoxycholate, produced primarily from bile acids by bacteria such as *Ruminococcaceae* and *Enterobacteriaceae* via 7a-dehydroxylated modifications, is a secondary bile acid that plays a role in limiting the proliferation of pathogens [1]. Gut microbes regulate antiviral immunity through secondary bile acids restoring the IFN signaling axis, thereby influencing virus transmission [72]. Deoxycholic acid also prevents COVID-19 by inhibiting cytokine burst and viral binding to angiotensin-converting enzyme inhibitor 2 [73]. Not only that, in in vitro studies, secondary bile acids inhibited *C. difficile* spore growth, and the restoration of secondary bile acids in the intestine contributed to the recovery of human colonization resistance to *C. difficile* [74]. It has also been suggested that the ratio of fecal deoxycholic acid to glycoursodeoxycholic acid is a strong predictor of recurrent *C. difficile* infection, and this ratio is reduced in most recurrences (84%) [75]. However, it is important to note that genetic data pertaining to these mechanism-related metabolites are currently lacking, and the metabolites discussed in this paper represent only a fraction of the entire spectrum of relevant compounds. Some of the metabolites with possible relationships in previous studies still failed to be included in the analysis. For example, acetate, propionate, and butyrate are short-chain fatty acids, mainly produced by the phylum *Bacteroidetes* and the phylum *Firmicutes*, that regulate host immunity and metabolism by interacting with cell-expressed G protein-coupled receptors [76]. Polyamines play a crucial role in promoting the synthesis of intercellular linker proteins, which are essential for regulating paracellular permeability and enhancing the integrity of the epithelial barrier [77]. Metabolic pathways were likewise identified in the menaquinol 8 biosynthesis II metabolic pathway to have a positive association with LRTIs. Menaquinol 8, a subtype of vitamin K2, is required for spore formation and cytochrome formation in some Gram-positive bacteria [78]. Vitamin K2 biosynthesis is associated with a variety of diseases and states, such as type 2 diabetes mellitus and Alzheimer’s disease [79,80]. A positive correlation has been observed between the vitamin K2 biosynthetic pathway and clinical prognosis in critically ill COVID-19 patients, suggesting a potential improvement in clinical outcomes [81].

We propose the hypothesis that the microbial abundance, microbial metabolites, and functional pathways, which were identified as playing a causal role in respiratory tract infections in this study, may be interconnected. Specifically, LRTIs might exhibit higher susceptibility to gut flora metabolites through the bloodstream. It is noteworthy that various gut microbes rely on the menaquinol 8 biosynthetic pathway. However, it should be taken into consideration that menaquinol 8, synthesized by intestinal bacteria, is rarely absorbed into the systemic circulation and remains undetected in the blood [82]. Consequently, the lung-related mechanism of menaquinol 8 may rely on the blood metabolite deoxycholate. Previous studies have provided evidence that the administration of exogenous vitamin K2 supplementation leads to an increase in fecal secondary bile acids and short-chain fatty acids in both diabetic patients and mice [83]. Further validation is required to elucidate the specific underlying mechanisms.

However, there are still some limitations in this paper. Firstly, some of the data selected in this paper were old, and the latest possible data were not retrieved. Secondly, there is a lot of variation from genes to phenotypes, and we manually removed the confounding bias associated with confounding factors whenever possible. However, confounding factors still existed, especially the unexplored. Finally, the populations included in this paper were from Europe, and further external validation for other populations is still needed.

## 5. Conclusions

Our study utilizing MR has successfully identified six bacterial groups that are associated with the incidence of respiratory infections. These findings not only provide valuable clues for future research but also highlight the potential for regulation from a single variable perspective. Additionally, we have uncovered microbial metabolites and pathways that exhibit causal relationships specifically with LRTIs. Notably, deoxycholate and the menaquinol 8 biosynthesis pathway have emerged as playing potential roles in infection development. The exploration of whether exogenous supplementation or inhibition of these factors can yield tangible benefits represents a promising avenue for future investigation.

In summary, our study not only expands our knowledge regarding the impact of the gut microbiota on respiratory infections but also underscores the potential for targeted interventions based on these findings. The identification of specific bacterial groups, metabolites, and pathways opens new avenues for therapeutic strategies. A comprehensive understanding of the underlying mechanisms will enable the development of effective interventions that can modulate the gut microbiota and ultimately reduce the incidence and severity of respiratory infections.

## Figures and Tables

**Table 1:** Summary results of the positive MR results and heterogeneity tests after correction.

Infections	Exposures	nSNP	MR Analysis						Cochran’s Q Test		MR Egger Intercept		Causal Direction *
			Method	b	SE	*p* Value	OR	95%CI	Value	*p* Value	Value	*p* Value	
URTIs	Family *Lactobacillaceae*	6	IVW	−0.118	0.039	0.002	0.889	0.824–0.959	2.958	0.706	−2.56 × 10^−3^	0.907	TRUE
			MR Egger	−0.103	0.129	0.470	0.903	0.701–1.162	2.942	0.568			
			Weighted median	−0.128	0.053	0.016	0.880	0.793–0.976					
			Simple mode	−0.141	0.076	0.122	0.868	0.748–1.007					
			Weighted mode	−0.138	0.066	0.092	0.871	0.765–0.992					
	Genus *Family XIII AD3011*	12	IVW	−0.136	0.046	0.003	0.873	0.798–0.955	9.950	0.535	−2.18 × 10^−2^	0.228	TRUE
			MR Egger	0.125	0.208	0.562	1.133	0.754–1.704	8.297	0.600			
			Weighted median	−0.076	0.064	0.234	0.927	0.818–1.05					
			Simple mode	−0.045	0.097	0.651	0.956	0.791–1.156					
			Weighted mode	−0.049	0.085	0.576	0.952	0.807–1.124					
LRTIs	Order *Bacillales*	7	IVW	0.001	0.001	0.020	1.001	1.000–1.002	5.924	0.432	−9.13 × 10^−5^	0.897	TRUE
			MR Egger	0.002	0.005	0.703	1.002	0.992–1.012	5.902	0.316			
			Weighted median	0.001	0.001	0.155	1.001	1.000–1.003					
			Simple mode	0.001	0.001	0.334	1.001	0.999–1.004					
			Weighted mode	0.001	0.001	0.307	1.001	0.999–1.003					
	Genus *Ruminococcaceae UCG009*	8	IVW	−0.003	0.001	0.002	0.997	0.996–0.999	2.343	0.938	2.96 × 10^−4^	0.739	TRUE
			MR Egger	−0.006	0.010	0.572	0.994	0.975–1.014	2.222	0.898			
			Weighted median	−0.002	0.001	0.037	0.998	0.996–1.000					
			Simple mode	−0.002	0.002	0.223	0.998	0.995–1.001					
			Weighted mode	−0.002	0.001	0.188	0.998	0.995–1.001					
	Genus *Paraprevotella*	7	IVW	0.003	0.001	0.001	1.003	1.001–1.004	2.593	0.858	−1.00 × 10^−4^	0.844	TRUE
			MR Egger	0.004	0.005	0.495	1.004	0.994–1.014	2.55	0.769			
			Weighted median	0.002	0.001	0.034	1.002	1.000–1.005					
			Simple mode	0.003	0.002	0.176	1.003	0.999–1.006					
			Weighted mode	0.003	0.002	0.156	1.003	0.999–1.006					
	Genus *Alistipes*	7	IVW	−0.004	0.001	0.001	0.996	0.993–0.998	3.617	0.728	1.69 × 10^−4^	0.750	TRUE
			MR Egger	−0.007	0.009	0.443	0.993	0.975–1.01	3.503	0.623			
			Weighted median	−0.005	0.002	0.010	0.995	0.992–0.999					
			Simple mode	−0.006	0.003	0.063	0.994	0.989–0.999					
			Weighted mode	−0.002	0.002	0.361	0.998	0.993–1.002					
LRTIs	Deoxycholate	11	IVW	−0.004	0.002	0.005	0.996	0.993–0.999	11.086	0.351	3.31 × 10^−4^	0.149	TRUE
			MR Egger	−0.012	0.005	0.042	0.988	0.978–0.998	8.596	0.475			
			Weighted median	−0.003	0.002	0.214	0.997	0.993–1.002					
			Simple mode	0.000	0.003	0.909	1.000	0.994–1.007					
			Weighted mode	−0.001	0.004	0.767	0.999	0.992–1.006					
LRTIs	Menaquinol 8 biosynthesis II	3	IVW	0.002	0.001	0.002	1.002	1.001–1.003	0.187	0.911	−1.83 × 10^−5^	0.980	TRUE
			MR Egger	0.002	0.003	0.645	1.002	0.996–1.008	0.186	0.667			
			Weighted median	0.002	0.001	0.017	1.002	1–1.003					
			Simple mode	0.002	0.001	0.159	1.002	1–1.004					
			Weighted mode	0.002	0.001	0.160	1.002	1–1.004					

* The “TRUE” results concerning the causal direction mean there is no reverse causation.

## Data Availability

All the relevant data for this article were obtained online. The GWAS data concerning gut microbiota abundance were retrieved from www.mibiogen.org, and the list of gut metabolites was obtained from http://bio-annotation.cn/gutmgene/home.dhtml (accessed on 22 May 2020) and https://hmdb.ca (accessed on 22 May 2020), supplemented with GWAS summary data from three related studies. The functional pathways were obtained from https://dutchmicrobiomeproject.molgeniscloud.org/ (accessed on 25 May 2020). The outcome indicator URTI was obtained from https://gwas.mrcieu.ac.uk/ (accessed on 18 May 2020), while the LRTI was obtained from the database website https://www.finngen.fi/fi (accessed on 18 May 2020).

## Supplementary Materials

The following supporting information can be downloaded at: https://www.mdpi.com/article/10.3390/microorganisms11082108/s1, Table S1: All the results for the initial positive microbiota abundance with an IVW *p* < 0.05.; Table S2: All the results for the initial positive microbial metabolites with an IVW *p* < 0.05.; Table S3: All the results for the initial positive microbial pathways with an IVW *p* < 0.05.
